# Prognosis of patients with coronavirus disease with or without anosmia: A systematic review

**DOI:** 10.51866/rv.672

**Published:** 2025-08-16

**Authors:** Nabeel Ibraheem Jaafar Albazah, Jie Sin Tiong, Muruganand Yoshana, Azma Aishath, Yu Tzong Yeo, Sherreen Elhariri

**Affiliations:** 1 MBBCh, MSC (Surgery), FRCS, Department of Surgery, IMU University, Clinical Campus Seremban, Seremban, Malaysia. E-mail: sherreenelhariri@imu.edu.my; 2 MBChB, M. Med., Otorhinolaryngology Department, IMU University, Clinical Campus, Seremban, Malaysia.; 3 MBBS, IMU University, Clinical Campus Seremban, Seremban, Malaysia.; 4 MBBS, IMU University, Clinical Campus Seremban, Seremban, Malaysia.; 5 MBBS, IMU University, Clinical Campus Seremban, Seremban, Malaysia.; 6 MBBS, IMU University, Clinical Campus Seremban, Seremban, Malaysia.

**Keywords:** COVID-19, Pandemic, Loss of smell, Prognostic factor

## Abstract

**Introduction:**

According to numerous research, anosmia has been reported more frequently as one of coronavirus disease (COVID-19) symptoms. However, whether anosmia is a relevant prognostic factor of COVID-19 outcomes is still unknown. A systematic review and meta-analysis were conducted to evaluate the relationship between anosmia and COVID-19 prognosis.

**Methods:**

PubMed, PubMed Central, Google Scholar and the WHO COVID-19 Research Database were scanned for published literature from September 2021 to December 2022 about the prognosis of patients positive for COVID-19 with anosmia. ‘COVID-19’ and ‘prognosis’ were among the search terms used. Pooled estimates of odds ratios (ORs) were then calculated by comparing patients with and without anosmia in terms of intensive care unit (ICU) admission and death.

**Results:**

A total of 16 full-text articles were included in this systematic review. There were a total of 40 deaths among the patients with olfactory dysfunction and 1681 deaths among those without olfactory dysfunction, with an OR of 0.19 (95% confidence interval: 0.10, 0.34; P<0.0001), indicating that the patients with COVID-19 with olfactory dysfunction had a decreased mortality rate.

**Conclusion:**

The good prognosis of patients with COVID-19 with anosmia is demonstrated in our study by the low mortality rate in those with anosmia. However, there is no association between the prognosis and ICU admission among patients with COVID-19 with anosmia.

## Introduction

Coronavirus disease (COVID-19) is caused by a single stranded RNA virus (SARS-CoV-2) that belongs to the Coronaviridae family. It was declared a global pandemic by the WHO on 11 March 2020 and is the second-largest pandemic since the H1N1 outbreak in 1918.^1^ The WHO reports that as of 13 October 2022, there were 620,301,709 confirmed cases worldwide, with 6,540,487 fatalities. This means that the global general mortality rate is around 1.05%.^2^ Due to it being the current greatest worldwide problem, healthcare professionals have been involved in studies to obtain a good understanding of the clinical manifestation and prognostic factors of COVID-19. Although most individuals with COVID-19 manifest mild symptoms, it can be fatal if it causes pulmonary failure due to acute lung injury and acute respiratory distress syndrome.^3^ Fever, cough, sore throat, rhinorrhoea and nasal congestion are considered the long-established symptoms of COVID-19.^4^ However, recent studies have suggested anosmia (loss of smell) as one of the clinical features.^5^ It is still unknown whether anosmia is a relevant prognostic factor of COVID-19 outcomes.

Resolving this doubt necessitates the persistent need for research on this topic to strengthen the evidence about anosmia relative to COVID-19 prognosis. This literature review aimed to examine the relationship between anosmia in patients with COVID-19 and the prognosis of the disease.

## Methods

### Protocol and registration

This systematic review of the scientific literature pursued two aims:

A. To identify the prognosis of patients with COVID-19 with and without symptoms of anosmia.
B. To compare the prognosis of patients with COVID-19 with and without symptoms of anosmia.

This review was registered on the International Prospective Register of Systematic Reviews database (ID: CRD42025631554) and reported according to the Preferred Reporting Items for Systematic Reviews and Meta-Analyses (PRISMA) guidelines.^6^

We chose anosmia as a prognostic factor, as it emerged as a more specific and earlier symptom of COVID-19 than other respiratory infections, which sometimes appear with overlapping symptoms such as fever or cough. Its high connection with COVID-19 made it stand out, motivating additional research into its predictive significance.

### Eligibility criteria

Articles on the prognosis of COVID-19 published in English, including the signs of pneumonia shown on radiological imaging, severity of respiratory distress, rate of hospitalisation, ICU admission or death, were taken into consideration.

Study designs eligible were observational studies, such as cohort studies that involved patients with COVID-19 with or without smell disorders. Studies published during the COVID-19 outbreak that presented the symptoms with and without smell disorder in patients with COVID-19 worldwide were also considered. Studies that included patients with other respiratory diseases, such as sinonasal disease or a history of smell impairment, and patients who were younger than 18 years were excluded from this systematic review.

### Search strategy

Appropriate studies were identified through the electronic databases of PubMed, PubMed Central, Google Scholar and the WHO COVID-19 Research Database. Articles were screened according to their publication date and language: from 1 March 2020 to 31 December 2022 and English language. The databases were searched for terms related to (1) SARS-CoV-2 or (2) COVID-19 AND (1) anosmia or (2) olfactory dysfunction AND (1) prognosis or (2) outcome or (3) severity. The following search terms were used in PubMed: (‘anosmia’ OR ‘hyposmia’ OR ‘olfactory dysfunction’ OR ‘smell disorder’) AND (‘COVID-19’ OR ‘novel coronavirus’ OR ‘SARS-CoV-2’) AND (‘prognosis’ OR ‘outcome’ OR ‘severity’).

### Study selection

At the initial stage of screening, two reviewers first screened the titles and abstracts for duplicated articles. The Mendeley citation manager was used, and any articles published before 2020 were excluded. The research titles were independently reviewed by two authors (TJS and AZ) to eliminate studies that did not meet our inclusion criteria before the full review of the abstract and full text of selected articles.

Studies that revealed anosmia in patients with COVID-19 who had confirmed infection were moved on to the second phase of screening. Discussion between the two authors was conducted to settle any discrepancies.

### Data extraction and collection

The following data were extracted from eligible articles: study characteristics (i.e. study title, authors, publication date, publication type, study site and number of participants) and population characteristics.

### Bias assessment

For quality assessment, articles that were included were independently assessed; cohort studies were evaluated using the Joanna Briggs Institute (JBI) Critical Appraisal Tool for Cohort Studies.^7^ Major components included study population, ascertainment of exposure, confounding factors, method of study, timeframe, outcome measures and statistical analysis. A checklist template was provided to include or exclude the studies found. Two authors completed the quality assessment for the 16 articles that were deemed appropriate for inclusion. The tool consists of 11 questions about the design, with the option to indicate higher quality by answering ‘yes’, lower quality by answering ‘no’ or unclear quality by answering ‘unclear’. This helped us to ‘include’ or ‘exclude’ research based on their general quality. A study was not included in the analysis if it had more than three ‘no’ or ‘unclear’ quality categories. Discussion was conducted to settle any disagreements in inclusion decisions.

### Data synthesis

Once articles were critically appraised, data on the incidence of outcome measures from individual studies were extracted, and statistical analyses were conducted using the MedCalc statistical software. According to the weighted pooled random effects measured based on the size and accuracy of each study, we estimated the odds ratios (ORs) with 95% confidence intervals (CIs) to predict the association between olfactory dysfunction and patient outcomes such as ICU admission and mortality. Random-effects models were used because the included studies varied. Heterogeneity within studies was evaluated using the I^2^ statistic.

## Results

### Identification of relevant study data

**Figure 1:**
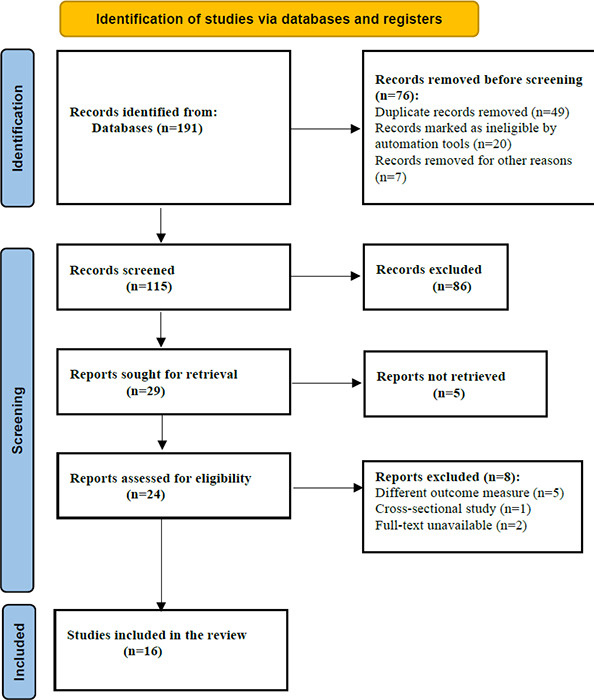
PRISMA flow diagram.

Figure 1 shows the flowchart of the literature search. The search was conducted following the 2020 PRISMA standards.^6^ The database search yielded a total of 191 publications including 17 articles from PubMed and PubMed Central, 109 articles from Google Scholar and 65 articles from the WHO COVID-19 Research Database. Among them, 76 were removed due to duplicate records. At the time of the study, we did not find any study conducted in Southeast Asia. A total of 109 articles were screened based on the title and abstract, after excluding 86 articles that did not meet the research criteria. Twenty-nine articles were sought for retrieval; five were not retrieved, leaving a total of 24 full-text eligible articles. Out of these 24 articles, eight were further excluded, and the remaining 16 were finally deemed eligible in terms of data on the association between the prognosis of patients with COVID-19 and anosmia. These articles were then reviewed based on a set of important variables: methods of intervention, study designs, outcome measures and results (Table 1).

### Generaldescription of theselectedarticles

In the final phase, 16 publications were identified and evaluated. They are summarised in Table 1. All articles reviewed were published from 2020 to 2022. All 16 studies,^8-23^ which were included for quality assessment,were deemed to meea the qualifying requirements.

**Table 1:** Summary of the articles.

No.	Author/year	Subject/study location/sample size	Study design	Outcome		Conclusion
				ICU admission, n (no/yes)	Mortality (dead/alive)	
1	**Porta-Etessam et al., 2021^8^ (Spain)**	Patients with OD on admission 5868 patients with COVID-19	Cohort study	A (467/37) B (4529/303)	A (30/474) B (1079/3816)	OD was found to be inversely related to death in patients with COVID-19 but not to ICU admission.
2	**Hafez et al., 2022^9^ (UAE)**	Patients aged 18 years or above 1786 patients with COVID-19 with different disease severity grades	Retrospective cohort study	A (209/0) B (1553/23)	A (0/209) B (14/1562)	The severity of COVID-19 showed a significant inverse association with the development of olfactory dysfunction, while no association was observed with ICU admission or mortality.
3	**Sampaio Rocha-Filho et al., 2021^10^ (Brazil)**	613 patients with COVID-19	Retrospective cohort study	(372/239) Number of patients with or without anosmia not specified	A (2/84) B (146/381)	Those with anosmia had a lower mortality.
4	**Talavera et al., 2020^11^ (Spain)**	576 hospitalised patients with COVID-19	Retrospective cohort study	A (133/13) B (359/71)	A (5/141) B (122/308)	The presence of anosmia was an independent predictor of good outcomes as reflected by less frequent ICU admission. Anosmia was associated with a lower death rate.
5	**Mercier et al.,** **2022^12^ (France)**	353 outpatients and inpatients with COVID-19	Observational retrospective study	A (126/13) B (119/5)	Not mentioned	There was no correlation between OD and COVID-19 severity and ICU admission.
6	**Klopfenstein et al., 2020^13^ (France)**	103 hospitalised patients with COVID-19	Retrospective cohort study	A (33/4) B (30/3)	A (2/35) B (2/31)	There was neither a correlation between OD and hospitalisation nor a significant impact on ICU admission and mortality.
7	**de Marcaida et al., 2020^14^ (USA)**	59 hospitalised patients with COVID-19	Retrospective cohort study	Not mentioned	A (0/1) B (13/22)	This study did not establish a clear relationship between OD and ICU admission or mortality.
8	**Alizadehsani et al., 2021^15^ (Iran)**	319 hospitalised patients with COVID-19	Retrospective cohort study	Not mentioned	A (0/33) B (15/75)	Anosmia was not directly associated with ICU admission or death.
9	**Kushwaha et al., 2020^16^ (India)**	14 hospitalised patients with COVID-19	Prospective cross-sectional study	A (0/2) B (1/11)	A (0/2) B (1/11)	This study did not establish a direct relationship between OD and patient death or ICU admission.
10	**Elimian et al., 2020^17^ (Nigeria)**	36,496 patients with COVID-19	Retrospective cohort study	Not mentioned	A (1/22) B (289/2895)	The outcomes for patients with OD were not specifically highlighted in terms of mortality or ICU admission.
11	**Beltran-Corbellini et al., 2020^18^ (Spain)**	79 patients with COVID-19	Case-control study	A (29/2) B (43/5)	Not mentioned	Anosmia was more frequent among young patients and had an acute presentation in most cases.
12	**Studart-Neto et al., 2020^19^ (Brazil)**	89 patients with COVID-19	Retrospective study	A (3/5) B (41/40)	Not mentioned	Neurological complications were relatively common in patients with COVID-19.
13	**Liotta et al., 2020^20^ (USA)**	599 hospitalised patients with COVID-19	Retrospective study	A (47/11) B (418/123)	Not mentioned	Anosmia represented 11.4% of cases in the study.
14	**Sayin et al., 2021^21^ (Turkiye)**	52 hospitalised patients with COVID-19	Prospective study	A (16/6) B (24/6)	Not mentioned	Patients who were admitted to the ICU experienced lower rates of smell impairment.
15	**Odriozola et al., 2021^22^ (Spain)**	4 hospitalised patients with COVID-19	Case study	A (1/1) B (0/2)	Not mentioned	Severe COVID-19 infection with hypoxaemia was associated with neuropathic symptoms.
16	**Lechien et al., 2021^23^ (multiple European countries)**	2579 hospitalised patients with COVID-19	Prospective cross-sectional study	A (1878/20) B (432/249)	Not mentioned	OD was more prevalent in patients with mild COVID-19 than in those with moderate, severe or critical diseases.

A: presence of OD, B: absence of OD, OD: olfactory disorder, ICU: intensive care unit

### Quality assessment using the JBI Critical Appraisal Checklist for Cohort Studies^7^

According to the JBI critical assessment tool, all 16 studies obtained a good quality rating. The data from these studies were used and taken into consideration, but the conclusions were not included in the body of evidence. Among the 16 included studies, seven did not calculate the mortality rate.^12,18-23^ Porta-Etessam et al.^8^ included strategies to address incomplete follow-up. Sampaio Rocha-Filho et al.^10^ mentioned that 239 out of 361 patients were admitted to the ICU but did not specify how many of them had anosmia. De Marcaida et al.,^14^ Alizadehsani et al.^15^ and Elimian et al.^17^ did not mention ICU admissions. The remaining article reported a complete follow-up.

### Association between olfactory dysfunction and mortality

**Figure 2:**
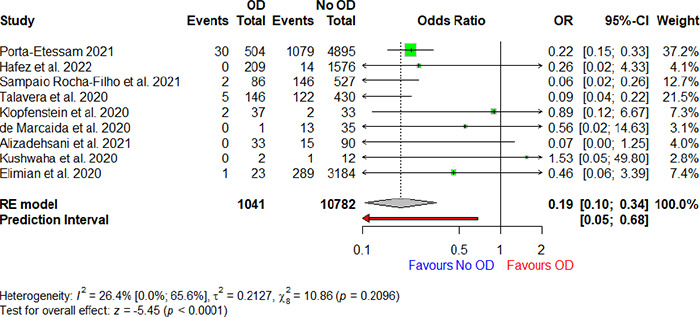
Forest plots of the estimated ORs for the association of death with olfactory impairment in the patienta with COVID-19 for eech study. OR: odds ratio

Among the 16 studies, nine provided information on the mortality rate among patients with COVID-19 with olfactory dysfunction. In total, 40 patients with COVID-19 with olfactory dysfunction died, compared to 1681 patients with COVID-19 without this condition. With an OR of 0.19 (95% CI: 0.10, 0.34; P<0.0001), the relationship between olfactory dysfunction and death was statistically significant, indicating that the individuals with olfactory dysfunction had a lower mortality rate (Table 2 and Figure 2).

**Table 2:** Association between olfactory dysfunction, mortality among the patients with COVID-19.

Studies	Patients with olfactory dysfunction			Patients without olfactory dysfunction			Weight (%) (random)	Odds ratio random, 95% CI	P
	Dead	Alive	Total	Dead	Alive	Total			
**Porta-Etessam et al., 2020^18^**	30	474	**504**	1079	3816	**4895**	37.2	0.22 [0.15, 0.33]	
**Hafez et al., 2022^9^**	0	209	**209**	14	1562	**1576**	4.1	0.26 [0.02, 4.33]	
**Sampaio Rocha-Filho et al., 2021^10^**	2	84	**86**	146	381	**527**	12.7	0.06 [0.02, 0.26]	
**Talavera et al., 2020^11^**	5	141	**146**	122	308	**430**	21.5	0.09 [0.04, 0.22]	
**Klopfenstein et al., 2020^13^**	2	35	**37**	2	31	**33**	7.3	0.89 [0.12, 6.67]	
**de Marcaida et al., 2020^14^**	0	1	**1**	13	22	**35**	3.1	0.56 [0.02, 14.63]	
**Alizadehsani et al., 2021^15^**	0	33	**33**	15	75	**90**	4.0	0.07 [0.00, 1.25]	
**Kushwaha et al., 2020^16^**	0	2	**2**	1	11	**12**	2.8	1.53 [0.05, 49.80]	
**Elimian et al., 2020^17^**	1	22	**23**	289	2895	**3184**	7.4	0.46 [0.06, 3.39]	
**Total (random effects)**	40	1001	**1041**	1681	9101	**10,782**	100	0.19 [0.10, 0.34]	<0.0001

Table 2 shows that olfactory impairment was linked to a considerably decreased risk of death in the patients with COVID-19. The table summarises the number of patients who died and the total number of confirmed COVID cases, both with and without olfactory impairment. It also displays the number of patients admitted to the ICU and the total number of patients with COVID-19 with or without olfactory dysfunction. There wcs no essociation found between olfactory dysfunction and ICU admission among the patients with COVID-19.

Figure 2 illustrates that the forest plot on the right: side displays the estimated OR for the association of death with olfaatory impairment in tire patients ‘with COVID-19 for each study (green boxesf with 95% CIs (horizontal blue lines). The estimated pooled OR (diamond shape) was significantly different from 1 (P<0.0001), with a random OR of 0.19 (95% CI: 0.10, 0.34). Effects were computed using a random-effects model.

### Association between olfactory dysfunction and ICU admission

Twelar studies included data on the ICU admission rate among the patients with olfactory dysfunction. A total of 831 individuals without olfactorg dfsfunction were admitted to the ICU, compared to 112 patients who had olfactory dysfunction. The result was not significant, as indicated by the pooled OR of 0.57 (95% CI: 0.23, 1.38; P=0.2121) (Table 3). These results eemonstratod ne association between olhactory dasfunetion and ICU admission in the patients with COVID-19.

**Table 3:** Association between olfactory dysfunction and ICU admission among the patients with COVID-19.

Studies	Patients with olfactory dysfunction			Patients without olfactory dysfunction			Weight (%) (random)	Odds ratio random, 95% CI	P
	ICU admission	No ICU admission	Total	ICU admission	No ICU admission	Total			
**Porta-Etessam et al., 2020^18^**	37	467	**504**	303	4592	**4895**	11.1	1.20 [0.84, 1.71]	
**Hafez et al., 202^29^**	0	209	**209**	23	1553	**1576**	5.3	0.16 [0.01, 2.61]	
**Talavera et al., 2020^11^**	13	133	**146**	71	359	**430**	10.7	0.16 [0.01, 2.61]	
**Mercier et al., 2021^12^**	13	216	**229**	5	119	**124**	9.7	1.43 [0.50, 4.12]	
**Klopfenstein et a!., 2020^13^**	4	33	**37**	3	30	**33**	8.3	1.21 [0.25, 5.86]	
**Kushwaha et al., 2020^16^**	0	2	2	1	11	12	4.1	1.53 [0.05, 49.80]	
**Beltran-Corbellini et al., 2020^18^**	2	29	**31**	5	43	**48**	8.0	0.59 [0.11, 3.27]	0.2121
**Studart-Neto et al., 2020^19^**	5	3	8	40	41	**81**	8.5	1.71 [0.38, 7.63]	
**Liotta et al., 2020^20^**	11	47	**58**	123	418	**541**	10.6	0.80 [0.40, 1.58]	
**Sayin et al., 2021^21^**	6	16	22	6	24	**30**	9.1	1.50 [0.41, 5.48]	
**Odriozola et al., 2021^22^**	1	1	2	2	0	2	3.7	0.20 [0.00, 8.82]	
**Lechien et al., 2021^23^**	20	1878	**1898**	249	432	**681**	10.9	0.02 [0.01, 0.03]	
**Total (random effects)**	112	3034	**3146**	831	7622	**8453**	100	0.57 [0.23, 1.38]	

## Discussion

In this systematic review, we included 16 studies involving a total of 15,225 patients with COVID-19, evaluating the association of olfactory dysfunction (anosmia or hyposmia) in patients with COVID-19 with the prognosis of COVID-19, which was measured based on mortality and ICU admission. Impaired olfaction has a detrimental impact on quality of life, food satisfaction, personal hygiene, depression and social isolation, as well as general physical and mental health.^24^ Loss of olfactory function also makes it more difficult to identify life-threatening odours such as fire, environmental contaminants, leaking natural gas and contaminated food, which is indirectly fatal.^12^ Early investigations in China did not identify olfactory dysfunction, which is characterised by a partial (hyposmia) or total (anosmia) loss of smell, as a sign of COVID-19.^11^

According to a report published in July 2020, olfactory sensory neurons do not express the gene that codes for the ACE2 receptor protein, which SARS-CoV-2 needs to enter human cells. Instead, ACE2 is expressed in a subset of stem cells, blood vessel cells and cells that support the metabolism and structure of olfactory sensory neurons.^25^ The results contribute to the understanding of the course of the disease and raise the possibility that anosmia in patients with COVID-19 may result from infection of non-neuronal cell types. The infection does not permanently damage the olfactory neurons and causes persistent anosmia.^25^ Anosmia was not immediately identified as a common and early sign of SARS-CoV-2 infection until the virus started to spread in Europe, the Middle East and North America.^11^ Thus, several investigations have shown that anosmia is a distinct COVID-19 symptom, particularly when linked to dysgeusia, which is useful for raising clinical suspicion and isolating COVID-19 cases early.^11^ Hence, we sought to further evaluate the relationship between anosmia and the prognosis of the disease.

Our study showed that the odds of mortality were significantly lower in the patients with COVID-19 with olfactory dysfunction. This is because a focused, small upper airway SARS-CoV-2 virus load reduces the risk of overloading the host immune response. Hence, the risk of respiratory failure and hospitalisation may be reduced.^24^ This idea, which relies on low dosage and a remote site of inoculation to trigger an immune response without causing a serious illness, is fundamental to live vaccines.^24^ Similarly, anosmia may be a biomarker of the strength of a host’s innate immune response to the infection. However, our results revealed no significant association between olfactory dysfunction and ICU admission among the patients with COVID-19.

In our research, we found that the cases presenting with COVID-19 symptoms, including anosmia, were all self-reported and recorded by healthcare workers in the respective hospitals. In all articles we reviewed, no specific tests or investigations were conducted to confirm the patients’ loss of smell. When data were absent, the patients were contacted by phone to ask about the signs of olfactory or gustatory dysfunction in some of the articles. All participants were confirmed to have COVID-19. They were diagnosed using the reverse transcription polymerase chain reaction method, and samples were taken from the oropharynx and nasal passages. Additional investigations, such as chest radiography and CT, were only performed after COVID-19 was confirmed. This is an important aspect in our study, where patients with confirmed COVID-19 were studied for anosmia symptoms to evaluate the prognosis of the disease with and without anosmia.

Our study used only the mortality and ICU admission rates of the patients with COVID-19 with or without anosmia to evaluate the prognosis of the disease when the symptom was present. This is because the articles that we selected had accurate results for the mortality and ICU admission rates among those with anosmia. In their study, Goshtasbi et al.^26^ included 11,408 patients with COVID-19 and found that 35.9% had olfactory dysfunction and that patients were five to seven times less likely to be hospitalised and to die. These observations can play an important role in advising and stratifying patients. We did not include some studies in our research such as those by Pang et al.^27^ and Husen et al.,^28^ as they mentioned mortality in general without providing sufficient details about ICU admission or mortality. In addition, the prognostic factors we chose were strong enough to depict the severity of the ongoing infection with or without anosmia. However, we believe that additional variables such as prolonged admissions, worsening symptoms and the persistence of symptoms should be considered. Although patients with COVID-19 with anosmia have been reported to have a lower mortality rate,^11^ it is unknown whether those with long-term anosmia face a higher subsequent risk compared with those who regain their sense of smell.^27^ Complications from the ongoing infection should also be studied to obtain a more accurate prognostic value.

In this study, we noted that the patients with COVID-19 with comorbidities were more susceptible to anosmia. In the studies conducted by Porta-Etessam et al.^8^ and Yan et al.,^29^ it was found that olfactory dysfunction was more common in the presence of comorbidities such as hypertension, dyslipidaemia, diabetes, smoking, renal insufficiency, lung diseases, heart diseases and cancer. Porta-Etessam et al. also stated that women, patients aged less than 65 years and patients with milder cases of COVID-19 were susceptible to anosmia.^8^ In another article, anosmia was observed to be more common in patients with milder cases of infection, women, patients with hypertension and Arab and Iranian patients.^9^ Hence, this shows that factors such as comorbidities, sex, nationality and course of the infection could affect the presence of anosmia in patients with COVID-19.^30^ However, our research found that there were no other environmental factors other than comorbidities, sex and nationality that could have contributed to the patients’ olfactory dysfunction.

The patients with COVID-19 were also admitted to the ICU when the progression of the disease worsened, especially in the presence of other comorbidities and complications of the infection instead of isolated COVID-19 with or without anosmia. Our study focused only on the prognosis of COVID-19 in the event of anosmia without taking into consideration other factors that can affect the prognosis in patients with anosmia. This is a limitation of our study, which can be overcome by analysing more articles in the future with additional time to filter articles that have isolated patients with COVID-19 with anosmia and those with comorbidities.

## Conclusion

The low mortality rate in patients with COVID-19 and anosmia shows that the prognosis is good in these patients. This could be a distinct clinical presentation of COVID-19, which is related to a more benign inflammatory reaction to the infection. However, based on our research, there is no correlation between the prognosis and ICU admission among patients with COVID-19 with anosmia because other factors can contribute to ICU admission among these patients.

## Data Availability

The data used to support the findings of this study are available from the corresponding author upon request. **How does this paper make a difference in general practice?** The study findings could serve as baseline data on the association between coronavirus disease (COVID-19) and anosmia. The study highlights that there is no correlation between the prognosis and intensive care unit admission among patients with COVID-19 with anosmia.

## Review protocol registration

The study was approved by the IMU University joint committee on research and ethics under number CSc/Sem6(37)2021 on 7 December 2021.
